# Single-Cell RNA Sequencing Identifies New Inflammation-Promoting Cell Subsets in Asian Patients With Chronic Periodontitis

**DOI:** 10.3389/fimmu.2021.711337

**Published:** 2021-09-08

**Authors:** Shu-jiao Qian, Qian-ru Huang, Rui-ying Chen, Jia-ji Mo, Lin-yi Zhou, Yi Zhao, Bin Li, Hong-chang Lai

**Affiliations:** ^1^Department of Oral and Maxillo-facial Dentistry, Shanghai Ninth People’s Hospital, Shanghai Jiao Tong University School of Medicine, Shanghai, China; ^2^National Clinical Research Center for Oral Diseases, Shanghai, China; ^3^Shanghai Key Laboratory of Stomatology, Shanghai Research Institute of Stomatology, Shanghai Jiao Tong University, Shanghai, China; ^4^Department of Immunology and Microbiology, Shanghai Institute of Immunology, Shanghai Jiao Tong University School of Medicine, Shanghai, China; ^5^Department of Thoracic Surgery, Shanghai Pulmonary Hospital, Tongji University School of Medicine, Shanghai, China

**Keywords:** single-cell sequencing, gingiva, periodontitis, inflammatory microenvironment, mucosa

## Abstract

Periodontitis is a highly prevalent chronic inflammatory disease leading to periodontal tissue breakdown and subsequent tooth loss, in which excessive host immune response accounts for most of the tissue damage and disease progression. Despite of the imperative need to develop host modulation therapy, the inflammatory responses and cell population dynamics which are finely tuned by the pathological microenvironment in periodontitis remained unclear. To investigate the local microenvironment of the inflammatory response in periodontitis, 10 periodontitis patients and 10 healthy volunteers were involved in this study. Single-cell transcriptomic profilings of gingival tissues from two patients and two healthy donors were performed. Histology, immunohistochemistry, and flow cytometry analysis were performed to further validate the identified cell subtypes and their involvement in periodontitis. Based on our single-cell resolution analysis, we identified HLA-DR-expressing endothelial cells and CXCL13^+^ fibroblasts which are highly associated with immune regulation. We also revealed the involvement of the proinflammatory NLRP3^+^ macrophages in periodontitis. We further showed the increased cell-cell communication between macrophage and T/B cells in the inflammatory periodontal tissues. Our data generated an intriguing catalog of cell types and interaction networks in the human gingiva and identified new inflammation-promoting cell subtypes involved in chronic periodontitis, which will be helpful in advancing host modulation therapy.

## Background

Periodontitis, a chronic inflammatory lesion of the collective periodontium, is characterized by irreversible and progressive degradation of the periodontal tissue and causes tooth loss and alveolar bone defects. Accumulating evidence has linked periodontitis to some noncommunicable diseases including cardiovascular disease, diabetes, chronic kidney disease, respiratory diseases, and cognitive disorders (1, 2). Being the sixth most common human disease, severe periodontitis represents a substantial health and socioeconomic burden due to its health impact and high costs of treatment (3). However, current periodontal treatment approaches focusing primarily on biofilm reduction have shown insufficiency to result in clinical improvement and to prevent the relapse of the disease.

It is now well recognized that periodontitis results from dysbiosis and the dysregulation of immune homeostasis (4). Several types of cells are included in the hosts’ armamentarium against dysbiosis, epithelial cells, endothelial cells, fibroblast cells, immune cells, and undifferentiated mesenchymal cells (5–7). All these cells precisely orchestrate an appropriate response to the biofilm microorganism and its components. In periodontitis, however, an inappropriate and excessive host response arises, resulting in collateral periodontal tissue damage.

Considering the importance of host immune response in the pathogenesis of periodontitis, it is imperative to develop host modulation therapy. Recently, the diversity of cell subsets has been recognized as a substrate for host modulation strategies. For instance, the Treg-recruiting formulation system has been injected to treat severe experimental periodontitis (8). Agents inducing the inflammatory-to-resolving conversion of macrophages were suggested to arrest periodontitis progression and stimulate bone regeneration (9). It should be noted that the behavior of cells, especially immune cells, is highly regulated as the cells perceive the changes in the microenvironment (10, 11). However, these altered phenotypes finely tuned by the pathological microenvironment in periodontitis remained largely unknown.

Transcriptome analysis based on bulk tissue RNA-seq has provided comprehensive overview of molecular events of the inflammatory response in periodontitis (12–14). However, most studies only focused on the averaged transcriptional signatures on a preselected cell type or crossed all cell types in the whole tissue without the information on the cellular heterogeneity in the periodontal tissue. The advance in single-cell technologies offers an opportunity to obtain the transcriptomes of individual cell types in the human tissues (15, 16). Recent transcriptional profiling of oral mucosa by single-cell RNA sequencing had led to the identification of a stromal-neutrophil axis in tissue immunity (17) and a decrease of epithelial and mesenchymal subpopulations from health to mild oral inflammation (18).

So far, the key inflammation-promoting cell subsets and the interactive networks within the pathological microenvironment in periodontitis remain incompletely understood. Moreover, due to the higher susceptibility of Asians to severe periodontitis (19, 20), transcriptomic data are needed to better understand the host response in Asian patients. Herein, we investigated the inflammatory response within the periodontal microenvironment, based on the transcriptomic profiling of a total of 29,967 single cells of human gingival tissues from two Asian patients with periodontitis and two healthy donors. We identified that HLA-DR-expressing endothelial cells and CXCL13^+^ fibroblasts were highly associated with immune regulation. In addition, the immune cell proportion changed significantly in the inflammatory environment. The data indicated that the proinflammatory NLRP3^+^ macrophages play an important role in periodontitis and the increased cell-cell communication between macrophage and T/B cells existed in the inflammatory periodontal tissues. Our findings offer a novel perspective on the periodontal inflammatory microenvironment and serve as a useful resource for developing host modulation therapy whereby adjuncts to mechanical debridement for managing chronic periodontitis.

## Methods

### Sample Collection and Ethics Approval

Collection of samples was approved by the Ethics Committee of Shanghai 9th People’s Hospital in China (SH9H-2019-T158-2). The experiments conformed to the principles of the Helsinki Declaration revised in 2008. A total of 20 individuals were involved in this study, including 10 periodontitis patients and 10 healthy volunteers (**Supplementary Table S1B**). For periodontitis, only individuals with stage III or IV periodontitis according to the new classification of periodontitis (21) were enrolled. For periodontally healthy volunteers, individual with intact periodontium without clinical inflammation were enrolled (22). The inclusion criteria for all participants were as follows: (a) no smoking; (b) no systemic disease; (c) not pregnant or breastfeeding; (d) no medication within the preceding 3 months; and (e) no periodontal therapy within the previous 6 months. All donors gave informed consent prior to participation into the study. Clinical assessment and biopsy sampling were conducted at the Department of Oral Implantology, Shanghai 9th People’s Hospital, Shanghai Jiaotong University, China. Healthy samples were collected from healthy volunteers during crown-lengthening procedure. The diseased samples were collected from patients during open-flap debridement. For scRNA-seq, periodontal tissues of two patients with periodontitis and two healthy individuals were collected. The demographic information and clinical parameters of the two groups are shown in **Supplementary Table S1**.

### Histology and Immunohistochemistry

Human periodontal soft tissue biopsies were stained with H&E for morphological evaluation. For fluorescent immunostaining, human periodontal soft tissue biopsies were incubated with primary antibodies diluted in 3% bovine serum albumin (BSA)/phosphate-buffered saline (PBS) overnight at 4°C. Primary antibodies used include decorin (1:50, ab175404, Abcam, Cambridge, UK), osteoglycin (1:50, sc-374463, Santa Cruz Biotechnology, Dallas, TX, USA), HLA-DR (1:50, ab92511, Abcam), CD11b (1:50, ab8878, Abcam), CD3 (1:50, ab135372, Abcam), CD19 (1:50, ab134114, Abcam), CXCL13 (1:100, PA5-47035, Invitrogen, Waltham, MA, USA), and CD31 (1:50, ab9498, Abcam). Next-day samples were incubated in Alexa-fluor 488, 594 Monkey anti-Mouse, Alexa-fluor 647 Monkey anti-Rabbit, or Alexa-fluor 488 Monkey anti-Goat secondary antibodies (Jackson ImmunoResearch, Jackson, PA, USA). Nuclei were counterstained with 4′,6-diamidino-2-phenylindole (DAPI). Images were acquired using Zeiss LSM 880. Samples were evaluated in a blinded fashion at two to three different levels of sectioning according to the staining extent and intensity.

### Preparation of Single-Cell Suspensions

Once the sample was retrieved, it was dissociated and processed for scRNA-seq immediately. Periodontal soft tissue samples were minced into small fragments of less than 1 mm^3^ by surgical scissors and dissociated into single cells in dissociation solution (2 mg/ml IV collagenase, 2 Units/ml Dispase II in Ca2^+^- and Mg2^+^-free HBSS) covered with tinfoil on a shaker (shaking speed of 200 rpm) at 37°C for 60 min; 0.1 μg/mL DNase I was added in the last 10 min. The dissociated tissue was filtered to ensure single-cell suspension using 100 and 40 μm cell strainers (Falcon) successively. Cells were subjected to red blood cell lysis for 10 min and centrifuged and resuspended (500 g, 10 min) twice. After resuspension in defined volumes of PBS + 0.4% BSA, 10 μl of the cell suspension was used for cell counting by an automated cell counter (Thermo Fisher, Waltham, MA, USA) to determine the concentration of live cells. Single-cell samples with final cell viability above 90% and final concentration of 600–1,200 cells/μl were stored on ice until further processing. The whole procedure was performed on ice whenever possible.

### Flow Cytometric Analysis

Single-cell suspensions were washed twice with ice-cold flow cytometry staining (FACS) buffer (2% FBS + 1 mM EDTA in PBS), incubated with blocking buffer (1:100, 564765, BD Bioscience, Franklin Lakes, NJ, USA) for 15 min at 4°C. For cell surface antigen staining, single-cell suspensions were stained with CD45 (1:100, 368511, BioLegend, San Diego, CA, USA) and CD11b (1:100, 101228, BioLegend) in the dark for 1 h on ice and then washed two to three times with FACS buffer. For intracellular cytokine detection, cells were fixed and permeabilized with fixation/permeablization buffer (eBioscience, Waltham, MA, USA) according to the manufacturer’s protocol. Cells were then stained with NLRP3 (1:100, IC7578S-100UG, Novus, St. Louis, MO, USA), C1QA (1:100, NB100-64597, Novus), and PRDM1 (1:100, 565276, BD Bioscience) antibodies. Data were acquired within 2 h after staining on an LSR Fortessa (BD Biosciences), and analysis was performed by using FlowJo software (Tree Star, Ashland, OR, USA). Calculations made in bar graphs were generated in GraphPad Prism.

### Single-Cell RNA Sequencing and Read Processing

Single cells from independent periodontal samples were captured in four batches using the 10× chromium system (10× Genomics). The cells were partitioned into Gel Bead-In-Emulsions and barcoded cDNA libraries, then prepared using the Chromium Single Cell 3′ library and Gel Bead Kit v3 (10× Genomics). Single-cell libraries were sequenced in 100 bp paired-end configuration using an Illumina NovaSeq and mapped to the GRCh38 human reference genome using the Cell Ranger toolkit (version 3.0.0). The preliminary data analysis generated a file containing a barcodes table, a genes table, and a gene expression matrix. Next, we obtained an overview website containing a considerable amount of information, such as number of cells, median number of detected genes, sequencing saturation, and sequencing depth.

### Filtering and Normalization of scRNA-seq Data

We installed R (version 3.5.1) and Seurat R package (version 3.1.5) for downstream analysis. First, the substantial background levels of ambient RNA in the single-cell suspension caused problems for subsequent analysis. Thus, we applied SoupX (version 1.4.5) for background correction. Next, for quality control of each matrix, lowly detected genes (<0.1% cells) and cells with a small number of genes (<350 genes) were discarded from the downstream analysis. We the filtered out unhealthy cells that generally have high mitochondrial mRNA loads (>20%) and high ribosome RNA loads (>40%). We found that different cell types expressed different numbers of genes, particularly between immune and nonimmune cells. Thus, we applied a slightly different criteria to remove supposed *ambient RNA contamination* (<1,700 UMI for healthy detected per cell) and potential double droplets (>6,500 genes for healthy and >4,000 genes for periodontitis tissues detected per cell). After the step above, we obtained 10,501 high-quality periodontitis cells (median UMI: 3,544; median 1,203 genes/cell) and 19,977 healthy periodontal cells (median UMI: 16,455; median 3,169 genes/cell).

### Dimension Reduction, Unsupervised Clustering, Annotation, and Visualization

The expression value of each gene was first normalized by TPM/10 and then log-transformed (NormalizeData function in Seurat with default parameters). Using the variation-stabilizing transformation (vst) method, the top 2,000 variable genes were selected in each matrix and were used as input for the “FindIntegrationAnchors” function. The four expression matrices were then integrated with the “IntegrateData” function. The integrated data were dimension reduced with principal component analysis (PCA; top 30 dimensions) first and then further reduced to two dimensions with UMAP which was also used to visualize the clusters. The nearest neighbors were defined among cells with KNN method (FindNeighbors), and cells were then grouped with Louvain algorithm (FindClusters in Seurat, resolution equal to 1.5; PCA:top 25 dimensions). Specifically, we removed one cluster, considered contamination, due to the coexpressed markers of plasma and epithelial cells (data not shown). Finally, we retained a total of 29,967 cells after stringent quality controls for further analysis, with 19,806 (66.09%) from healthy tissues and 10,161 from the periodontitis tissues. Annotation of the clusters was performed by checking known markers for cell types that potentially would exist in the sample, and some clusters were merged as they were annotated as a major cell type. Average expression levels of each subtype marker were calculated by AddModuleScore in Seurat with default parameters. For subclustering, cells from a major cell type were taken as the input. We performed dimension reduction, clustering, and annotation using the same method as described above.

### Detection of Differentially Expressed Genes

To obtain the DEG list of each cluster, only the genes expressed in more than 30% of that cluster were considered, and the expression in all other cells was used as background. For statistical test, we used the default Wilcoxon’s test implemented in Seurat. DEGs were defined as genes whose log fold change was over 0.2 compared with the background and with a *q*-value (FDR) smaller than 0.05.

### Gene-Set Enrichment Analysis

We conducted the gene-set enrichment analysis for DEGs of each cluster using clusterProfiler (23), GSVA, and GSEABase packages, with which the enriched GO biological process terms were calculated.

### SCENIC Analysis

SCENIC analysis was conducted as described previously (24). We used the pySCENIC package (version 0.10.3), a lightning-fast python implementation of the SCENIC pipeline. The differentially activated TFs of each subcluster were identified by the Wilcoxon’s rank sum test against all the other cells of the same cell type.

### Gene Cluster Associated With M1/M2 and Proliferation Phenotypes

The M1/M2 phenotype of each macrophage cell was defined as the mean expression of gene signatures (23, 25). CCL5, CCR7, CD40, CD86, CXCL9, CXCL10, CXCL11, IDO1, IL1A, IL1B, IL6, IRF1, IRF5, and KYNU were included in M1 macrophage signature. Furthermore, CCL4, CCL13, CCL18, CCL20, CCL22, CD276, CLEC7A, CTSA, CTSB, CTSC, CTSD, FN1, IL4R, IRF4, LYVE1, MMP9, MMP14, MMP19, MSR1, TGFB1, TGFB2, TGFB3, TNFSF8, TNFSF12, VEGFA, VEGFB, and VEGFC are the member of the signature of M2 macrophages. In addition, the proliferation genes include AURKA, BUB1, CCNB1, CCND1, CCNE1, DEK, E2F1, FEN1, FOXM1, H2AFZ, HMGB2, MCM2, MCM3, MCM4, MCM5, MCM6, MKI67, MYBL2, PCNA, PLK1, TOP2A, TYMS, and ZWINT.

### Profiling the Cell-Cell Communication in Healthy and Periodontitis Samples

The cell-cell communication was measured by quantification of ligand-receptor pairs among different cell types. Gene expression matrices and metadata with major cell annotations were used as input for the CellphoneDB or CellChat (26) software. The default CellPhoneDB database and parameters were used. Healthy and periodontitis data were computed separately. The cell-cell network was visualized with circlize (version 0.4.10).

## Results

### Single-Cell Profiling of Human Gingival Tissues in Healthy Donors and With Periodontitis

**Figure 1:**
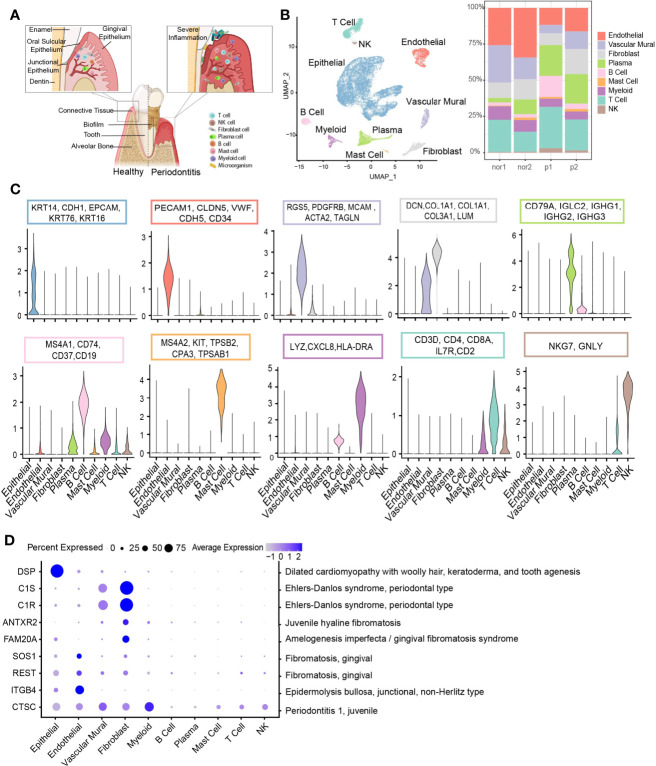
Overview of the clustering and annotation of the single-cell RNA sequencing data for gingival tissues. **(A)** Schematic of gingival tissues in health (left) and periodontitis (right) analyzed in this study (The graph was created with BioRender.com). **(B)** UMAP representation of major cell types identified by scRNA-seq (*n* = 4; 29,967 cells; left) and bar plots indicating the percentage of the nonepithelial of subtypes in each donor (Nor1 and Nor2 are healthy donors; P1 and P2 are periodontitis patients; right). **(C)** Violin plots showing the expression scores of selected canonical marker gene sets across all 10 subsets. **(D)** Dot plot depicting gene expression levels and percentage of cells expressing genes associated with periodontal disease according to the OMIM database.

To generate transcriptome profiles of human gingival tissues, samples from four Asian donors were obtained. Two of the donors were diagnosed with periodontitis (P1 and P2). The other two samples were obtained from healthy volunteers who underwent crown lengthening procedures (Nor1 and Nor2). Tissues were collected fresh, dissected, and digested into single cells (**Figure 1A** and **Supplementary Table S1A**). For each sample, single cells were captured using the droplet-based microfluidic chromium system (10× Genomics). The number of genes expressed differed in the various cell types, particularly between immune and nonimmune cells (**Supplementary Figures S1A, B**).

Considering potential batch effects and background noises among samples, we applied SoupX (27) to correct ambient RNA in the background and merged these data using the CCA method in Seurat (28). The single-cell data are presented in two-dimensional space using a uniform manifold approximation and projection (UMAP) method, and relative cell type abundance analysis within the stromal and immune cell population was performed (**Figures 1B**, **3F**). Clustering analysis cataloged these cells into 10 distinct cell lineages annotated with canonical marker gene expression, thereby corresponding to epithelial cells, stromal cells (endothelial, vascular mural, and fibroblast), and immune cells (T, NK, B, plasma, myeloid, and mast cells) (**Figure 1C**). Differential expressed genes (DEGs) of each cell type were computed and the top 5 DEGs were visualized (**Supplementary Figure S1C**).

Next, we assessed the cell-type-specific expression patterns of genes related to Mendelian disorders (based on OMIM database), which provided insights into the contribution of specific cell types to gingival abnormality (**Figure 1D** and **Supplementary Table S2**). Cell type-specific expression patterns confirmed fibroblast as particularly high expressors of the *C1S* and *C1R* gene, mutated in Ehlers-Danlos syndrome, periodontal type. Two reported gene mutants, *SOS1* (29) and *REST* (30), associated with gingival fibromatosis (GF), were highly expressed in endothelial cells. Additionally, the *CTSC* gene, mutations of which are responsible for aggressive periodontitis in juveniles, was found to be widely expressed in the nonimmune cell population. While in immune cells, the expression of *CTSC* was highly enriched in myeloid cells.

### Local Inflammatory Environment Within Human Gingival Tissue

**Figure 2:**
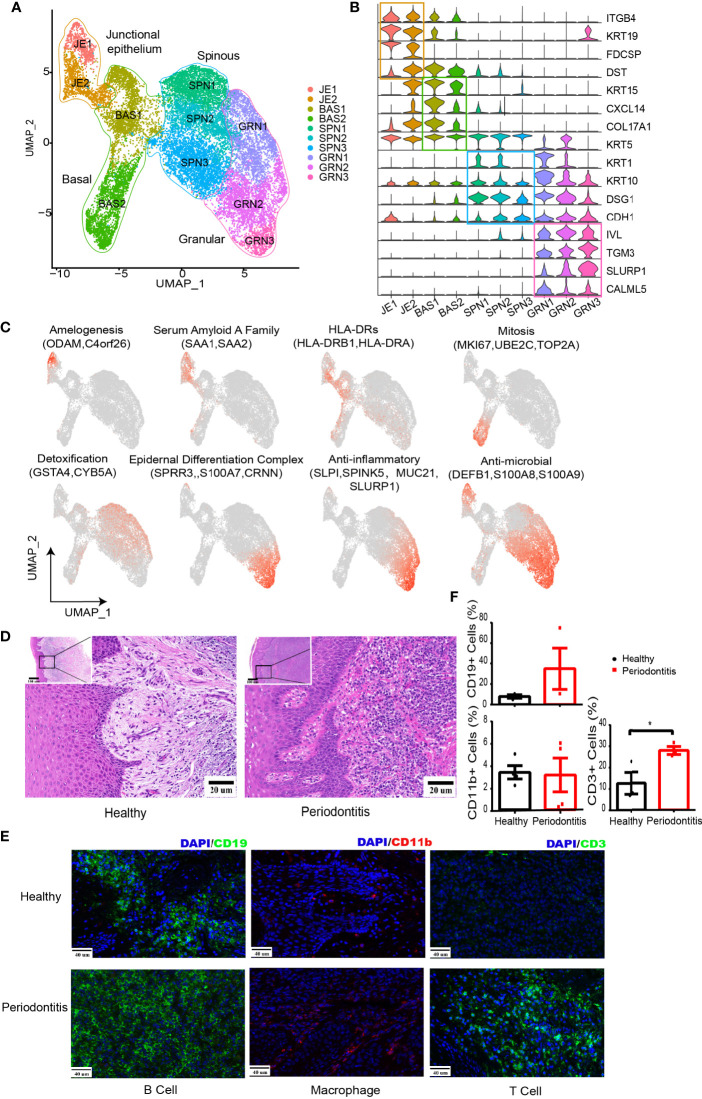
Changes in the microenvironment within human gingival tissue in periodontitis. **(A)** UMAP representation for human gingival epithelial cells in the combined health (*n* = 2; 18,012 cells) and periodontitis (*n* = 2; 502 cells) dataset. **(B)** Violin plots showing expression levels of cluster-defining genes for epithelial subsets. Expression values are normalized and scaled averages. **(C)** UMAP plots displaying functional molecule scores in the epithelial cells of all subpopulations. Red indicates maximum expression, and grey indicates low or no expression of each particular set of genes in log-normalized UMI counts. **(D)** H&E staining of the representative gingival tissue from health (left) and periodontitis (right) sections. Scale bar: 20 μm. **(E)** Representative immunofluorescence (IF) staining for CD19^+^ B cells (green, one column), CD11b^+^ macrophages (red, two columns), and CD3^+^ T cells (green, three columns) with DAPI (blue) in gingival sections of healthy (top) and periodontitis patients (bottom). Images were acquired at ×40 magnification (scale bar: 40 µm). **(F)** Bar graphs demonstrate percentage of CD19^+^ B cells (left top), CD11b^+^ macrophages (left bottom), and CD3^+^ T cells (right bottom) in gingival sections of healthy (*n* = 4) and periodontitis patients (*n* = 4) based on immunofluorescence analysis. The number of cells is counted based on five different limited areas on a slide from one sample then the mean was calculated. One dot per individual. Small horizontal lines indicate the mean (± s.d.). *p ≤ 0.05, as determined by Student’s *t*-test.

The gingival epithelium provides the first line of defense against chemical, physical, or microbial challenge by following a regulated scheme of differentiation that results in the integrity of epithelial barrier. We partitioned the epithelial cells into four diverse clusters based on previous reports (31, 32): junctional epithelium (JE1-JE2), basal (BAS1-BAS2), spinous (SPN1-SPN3), and differentiated granular keratinocytes (GRN1-GRN3) (**Figures 2A, B** and **Supplementary Figure S2B**).

**Figure 3:**
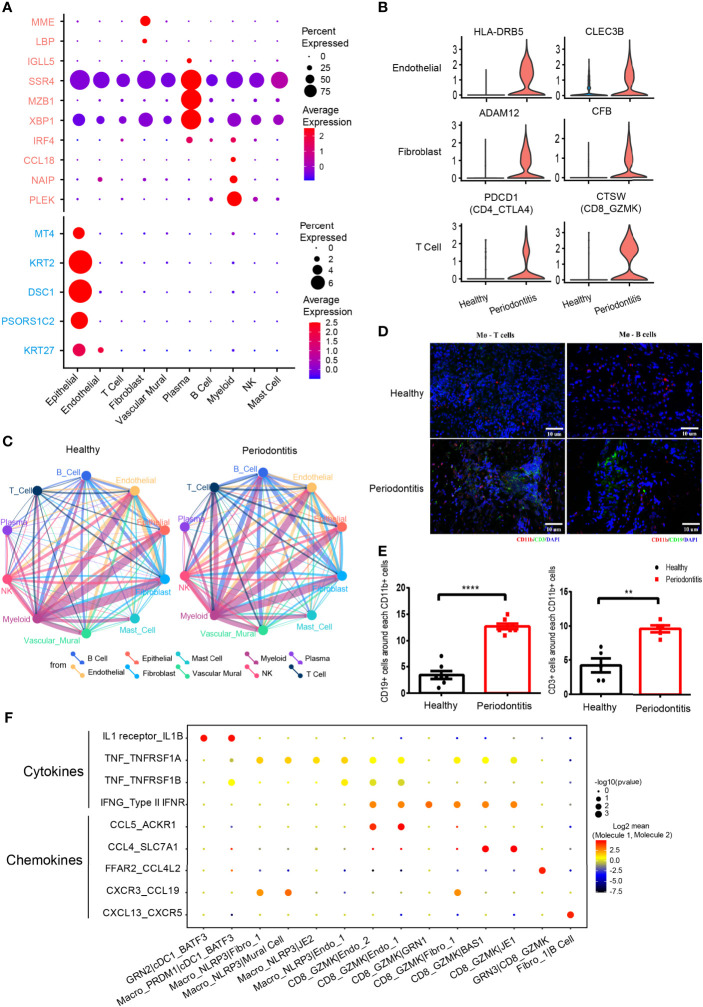
Differentially expressed genes and cell-cell interactions in health and periodontitis. **(A)** Bubble heatmap showing cellular expression patterns of genes associated with periodontitis based on previous bulk RNA sequencing. Rose stands for reported upregulated genes in periodontitis, and turquoise stands for reported downregulated genes in periodontitis. Dot size indicates fraction of expressing cells, colored according to z-score-normalized expression levels. **(B)** Violin plots showing the expression of specifically increased genes in different cell clusters in periodontitis. The gene expression levels are normalized and transformed as ln (CPM/10). **(C)** Cell-cell interaction network in health and periodontitis. Colors and widths of edges represent number of interaction pairs between cell types. **(D)** Immunofluorescent (IF) staining of T/B cell and macrophages. Red shows the signal of CD11b staining (macrophage marker); green shows the signal of CD3 (T-cell marker) at left and the signal of CD19 (B-cell marker) at right; and blue shows DAPI staining. **(E)** The number of CD3^+^ cells around each CD11b^+^ cell at left and the number of CD19^+^ cells around each CD11b^+^ cell at right (healthy donors, *n* = 5–7; patients, *n* = 5–7). The number of T/B cells within a limited distance of a macrophage cell is counted then the mean was calculated. One dot per individual. Small horizontal lines indicate the mean (± s.d.). **p ≤ 0.01, ****P < 0.0001 as determined by Student’s *t*-test. **(F)** Dot plot of the interaction between cytokine/chemokine and their receptors in selected subcell types of periodontitis. Size of spot indicates significance (−log10(*p*-value)). Color indicates expression levels (log2 mean (molecule 1–molecule 2).

We further analyzed the expression of characteristic genes and top 5 DEGs in each cluster (**Figure 2C** and **Supplementary Figure S2C**). JE1 expressed amelogenesis-associated proteins ODAM and ODAPH (C4orf26). Both JE1 and JE2 expressed serum amyloid A family (SAA1 and SAA2). Furthermore, HLA-DRs (HLA-DRB1 and HLA-DRA) presenting extracellular pathogens were found to be highly expressed in JE2 and BAS1. BAS2 exhibited high levels of mitosis-related genes, denoting its possible stage at G2/m stages (**Supplementary Figure S2D**). The three spinous clusters were found to express *GSTA4* involved in antioxidative stress (33) and *CYB5A* detoxifying carcinogens from cigarette (34). GRN2 and GRN3 expressed genes associated with the anti-inflammatory response. We also noticed that GRN1-3 and JE1 expressed β-defensins (*DEFB1*) against bacterial challenge (35) and *S100A7/8/9* modulating the inflammatory response (36).

Analysis of the pathway in epithelial subsets was performed by gene-set variation analysis (GSVA) (37) (**Supplementary Figure S2E**), supporting the above inference about the cell function, such as amelogenesis for JE1, DNA replication for BAS2, and keratinocyte differentiation for GRN3. Furthermore, SCENIC (38) was utilized to identify different transcription factors (TFs) underlying the regulation of each epithelial phenotype (**Supplementary Figure S2F**; **Supplementary Table S3**). For instance, RUNX2 might be a potential regulator of the ODAM expression in junctional epithelium.

Compared with the healthy tissue, all of the nonepithelial cell types have their proportion increased in periodontitis, including T and B cells, which is consistent with the inflammation phenotype. Hematoxylin and eosin (H&E) stain showed a dense infiltrate of lymphocytes in periodontitis tissue (**Figure 2D**). The increased infiltration of T and B cells in periodontitis were further confirmed with immunofluorescence analysis (**Figures 2E, F**). Furthermore, we combined our data with a recent published single-cell transcriptome profiling of human gingival tissue (18). A similar distribution with their data of healthy individual and mild periodontitis could be observed, which further confirms the reliability of our data (**Supplementary Figure S3A**).

### Endothelial and Fibroblasts Subclusters Highly Associated With Immune Regulation

To gain more insight into the local inflammatory microenvironment within the gingival tissue, next, we focused on stromal cell clusters.

**Figure 4:**
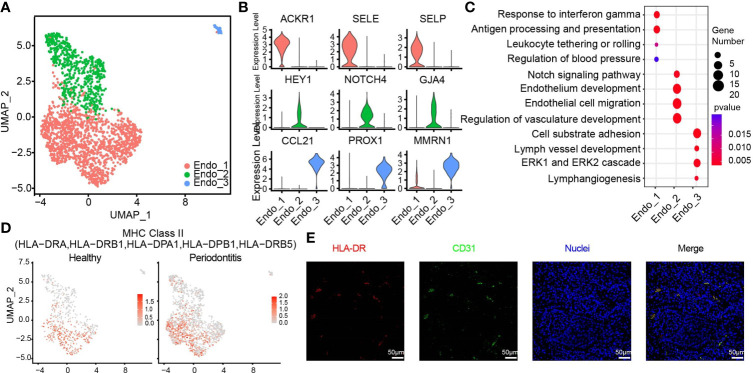
HLA-DR expressing endothelial subcluster in human gingiva. **(A)** UMAP visualization of endothelial subclusters in the combined health (*n* = 2; 596 cells) and periodontitis (*n* = 2; 1,363 cells) dataset. **(B)** Violin plots showing expression levels of cluster-defining genes for endothelial subsets. Expression values are normalized and scaled averages. **(C)** Dot plots showing the enriched gene ontology biological process terms for each endothelial cluster. **(D)** The expression of HLA-DR was projected on the UMAP plot. Red indicates maximum gene expression, while grey indicates low or no expression. The gene expression levels are normalized and transformed as ln (CPM/10). **(E)** Immunofluorescence assay for the MHC class II marker HLA-DR (red) and the endothelial cell marker PECAM1 (CD31, green) and with DAPI (blue) in the subepithelial region. Scale bar: 50 μm.

The 1,959 endothelial cells can be classified into three distinct subpopulations (**Figure 4A** and **Supplementary Figure S3B**). Endo_1 expressed *ACKR1*, *SELE*, and *SELP*. Endo_2 was characterized by *GJA4*, *HEY1*, and *NOTCH4*. We discovered a small group of lymphatic endothelial cells (LECs), named Endo_3, which expressed *PROX1*, *MMRN1*, and *CCL21* (39) (**Figure 4B**). The pathway analysis illustrates that Endo_1 is highly correlated with the immune response, response to interferon gamma, and upregulating adhesion molecules to tether or roll leukocyte. Meanwhile, Endo_1 is also involved in the regulation of blood pressure. In Endo_2, Notch signaling and other molecules contributing to endothelium development and migration were enriched. The enrichment pathways of Endo_3 meet the definition of LECs and are also involved in cell substrate adhesion (**Figure 4C**). We identified an endothelial cell state with high expression level of MHC class II genes such as *HLA-DRA*, *HLA-DRB1*, and *HLA-DPB1*, which was normally found in professional antigen-presenting cells (40). The endothelial cell states in healthy tissue, particularly Endo_1, exhibited lower feature score of MHC class II compared with those in diseased tissues, suggesting the importance of endothelial cells in gingival tissue-specific immunity (**Figure 4D**). Immunofluorescence assays for the MHC class II marker HLA-DR and the endothelial cell marker PECAM1 (CD31) further confirmed the existence of antigen-presenting endothelial cells in gingival tissues of periodontitis (**Figure 4E**).

**Figure 5:**
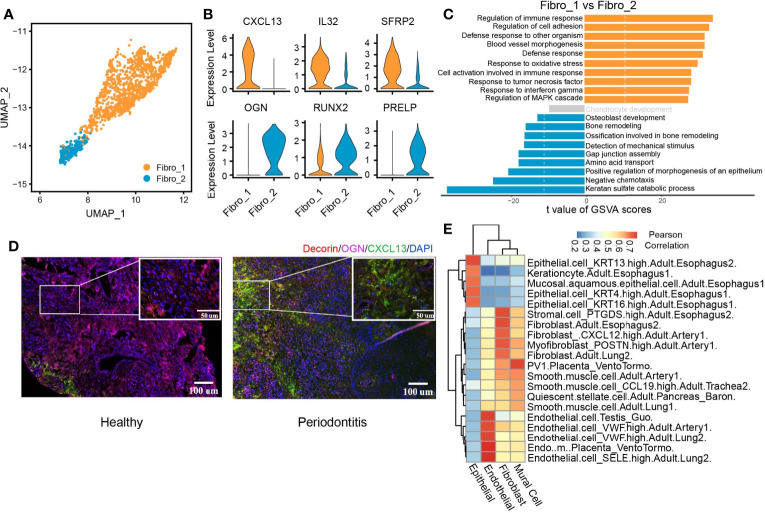
CXCL13^+^ fibroblast subcluster associated with immune response. **(A)** UMAP visualization of two fibroblast subclusters in the combined health (*n* = 2; 244 cells) and periodontitis (*n* = 2; 1,260 cells) dataset. **(B)** Violin plots showing the expression distribution of selected genes associated with functions in the fibroblast clusters. The gene expression levels are normalized and transformed as ln (CPM/10). **(C)** Differences in pathway activities scored per cell by GSVA between fibro_1 and fibro_2. Shown are *t* values from a linear model, corrected for fibro_1. **(D)** Immunofluorescent (IF) staining validation of fibroblast subtypes. Red shows the signal of decorin staining (fibroblast marker); green shows the signal of CXCL13 staining; purple shows the signal of osteoglycin (OGN), and blue shows DAPI staining. Scale bar: 100 μm for main images and 50 μm for detail images. **(E)** Application of scHCL analysis for nonimmune cells. Each row represents one cell type in scHCL. Each column represents a cell cluster in our dataset. Pearson’s correlation coefficient was used to evaluate cell-type gene expression similarity. Red indicates a high correlation; blue indicates a low correlation.

We identified two subclusters of fibroblasts with multiple differentially expressed genes against each other (**Figure 5A** and **Supplementary Figure S3C**). Fibro_1 was characterized by high expression of *CXCL13*, *IL32*, and *SFRP2*. Fibro_2 expressed higher levels of *OGN*, *PRELP*, and *RUNX2* compared with Fibro_1 (**Figure 5B** and **Supplementary Figure S3D**). We then compared pathway enrichment between two fibroblast clusters (**Figure 5C**). Fibro_1 showed increased immune response pathways. Pathways associated with osteoblast development and bone remodeling are increased in Fibro_2. The IF staining of the periodontitis gingival tissue also identified two fibroblast subpopulations, which is consistent with the seq data (**Supplementary Figure S3E**). Also, the predominant subpopulation converted from OCN^+^ fibroblasts (Fibro_2) to CXCL13^+^ (Fibro_1) fibroblasts when the gingival tissue was infiltrated with inflammatory cells in periodontitis (**Figure 5D**). Finally, we utilized scHCL (40) to verify the cluster identification and further explore the similarities between clusters (**Figure 5E**). By calculating Pearson’s correlation coefficients, the reliability of the cluster identification was confirmed. Interestingly, both epithelial and fibroblast cells were highly similar to those of esophageal origin, suggesting the effect of food intake on the cellular function of gingival fibroblast and epithelial cells.

### Diverse Immune Cell Subtypes With Hyperinflammatory Response in Periodontitis

**Figure 6:**
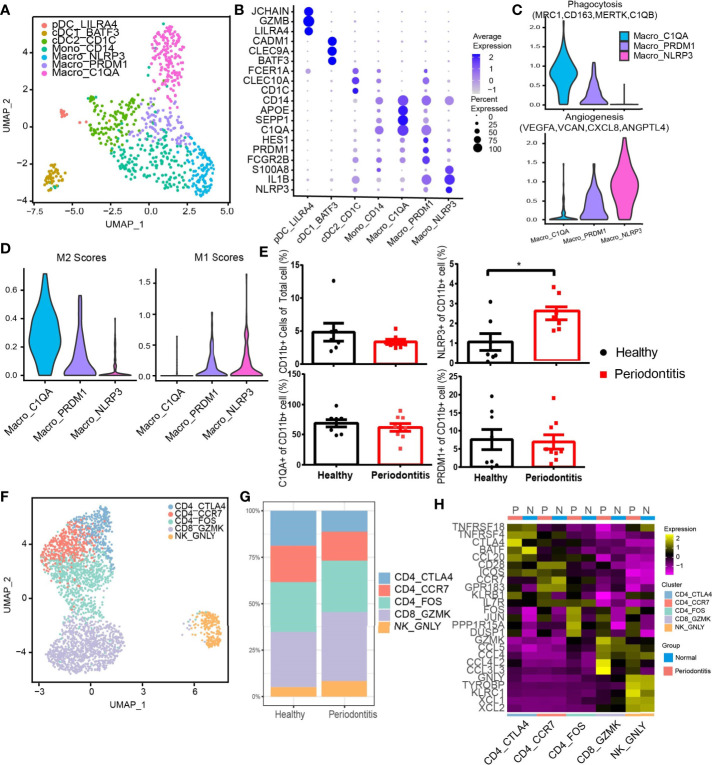
Diverse immune cell subtypes with hyperinflammatory response in periodontitis. **(A)** UMAP visualization of seven myeloid clusters in the combined health (*n* = 2; 148 cells) and periodontitis (*n* = 2; 539 cells) dataset. **(B)** Bubble heatmap showing marker genes across seven myeloid clusters from **(A)**. Dot size indicates fraction of expressing cells, colored according to z-score-normalized expression levels. **(C)** Violin plots showing the expression of angiogenesis- and phagocytosis-related genes (see *Methods* for details) in three macrophage clusters. The gene expression levels are normalized and transformed as ln (CPM/10). **(D)** Violin plots showing the expression of classically activated macrophages (M1) and alternatively activated (M2) macrophage-related genes (see *Methods* for details) in three macrophage clusters. The gene expression levels are normalized and transformed as ln (CPM/10). **(E)** The relative percentage of CD11b^+^ cells in total cells and the proportion of C1QA^+^, NLRP3^+^, and PRDM1^+^ subpopulations in CD11b^+^ cells were analyzed by flow cytometry (healthy donors, *n* = 8 and patients *n* = 8; see **Supplementary Figure S4B** for gating strategy). **(F)** UMAP visualization of five T and NK cell subclusters in the combined health (*n* = 2; 276 cells) and periodontitis (*n* = 2; 2,599 cells) dataset. **(G)** Bar plots indicate the relative proportion of T-cell subsets in each sample (healthy = 2 and periodontitis = 2). **(H)** Heatmap showing the average expression of the top 5 differentially regulated genes for T and NK clusters identified in healthy gingival and periodontitis tissues. *P < 0.05 as determined by Student’s t-test.

We next performed subclustering on myeloid cell types containing 148 cells from healthy donors and 539 cells from patients. We revealed seven subtypes of myeloid cells (**Figure 6A**). Three DC subsets were characterized by low expression of CD14, and three CD14-high expressing clusters were identified as macrophages based on their high expression of CD68, CD163, and MRC1 (24, 41) (**Supplementary Figure S4A**). Plasmacytoid DC (pDC), cDC1, and cDC2 were further distinguished by specific expression of *LILRA4/GZMB/JCHAIN*, *BATF3/CLEC9A/CADM1*, and *CD1C/CLEC10A/FCER1A*. Three clusters of CD14-high macrophages, Macro_PRDM1, Macro_NLRP3, and Macro_C1QA, were distinguished based on the expression of *FCGR2B/PRDM1/HES1*, *NLRP3/IL1B/EREG*, and *C1QA/SEPP1/SPOE*. There is a CD14^+^ monocyte cluster showing different features with DC and macrophages. All myeloid subtypes could be found in both normal and patient samples (**Figure 6B**). The pDC group highly expressed granzyme B, which has been reported to suppress T-cell expansion (42). Macro_NLRP3 exhibited itself as a proinflammatory phenotype by highly expressing NLRP3,a known inflammasome-mediating macrophage M1 polarization and interleukin (IL)-1β production in inflammatory diseases (43), as well as proinflammatory cytokine gene IL-1B and inflammatory biomarker gene S100A8 (44, 45). Macro_C1QA expressed SEPP1, a potential marker of anti-inflammatory M2 phenotype associated with antioxidant defense in the extracellular space (25, 46). Macro_C1QA also expressed a set of genes found previously in tumor-associated macrophages (47), including C1QA, APOE, and C1QB, resembling the signature of M2 phenotype (48).

The phenotypes of macrophages were analyzed in depth from angiogenesis and phagocytosis (**Figure 6C**). The gene associated with phagocytosis was highly expressed in Macro_C1QA, while Macro_NLRP3 dominated angiogenesis. Further analysis showed that Macro_PRDM1 simultaneously resembled the signatures of M1 and M2 cells (**Figure 6D**). Flow cytometry profiles also confirmed the three macrophage subpopulations in the gingival tissue and showed an increased proportion of CD11b^+^ NLRP3^+^ macrophages in gingival tissue in periodontitis patients compared with that in healthy individuals (**Figure 6E** and **Supplementary Figure S4B**), which suggested that the CD11b^+^ NLRP3^+^ subpopulation is highly associated with the development of periodontitis.

Another diverse immune cell cluster is T and NK cells, which were divided into five subtypes (**Figures 6F, G**). CD4_CTLA4 highly express Treg-associated molecules, including *TNFRSF18*, *TNFRSF4*, *CTLA4*, and *FOXP3* (**Figure 6H** and **Supplementary Figure S4C**). CD4_FOS from patients highly expressed immediate-early genes (*FOS*, *JUN*), which may be associated with T-cell activation or the effect of enzymatic digestion (49). Furthermore, CD8 and NK cells express cytotoxic genes, including *GZMK*, *GZMA*, *GNLY*, etc. Notably, an increased expression level of CCR5 or CCR1 ligand *CCL4/CCL4L2/CCL3L3* by CD8 T and NK cells was observed in patients.

### Differentially Expressed Genes and Cell-Cell Interactions in Healthy and Periodontitis Tissues

We investigated the cellular expression patterns of genes related to periodontitis, which were identified from previous bulk RNA sequencing (12–14, 50–53). We noticed that most of the genes upregulated in periodontitis were found to be expressed in plasma and myeloid cells. By contrast, genes downregulated in periodontitis were expressed in epithelial cells (**Figure 6A**). Differential expression analysis within individual cell types was performed (**Figure 6B**). In endothelial cells, HLA class II molecules *HLA-DRB5* and *CLEC3B* associated with extracellular proteolysis were upregulated in periodontitis. In fibroblast, a metalloprotease *ADAM12* and *CFB* involved in activating B cells was increased in disease. We also found *PDCD1* was highly upregulated in the CD4_CTLA4 cluster from periodontitis, which indicates that PD-1 pathway may contribute to the protective effect of Treg in disease stage (54). *CTSW* related to cytotoxic capacity was found to be upregulated in CD8_GZMK in periodontitis. These genes, previously masked in the mean expression data, provided novel insights for the characterization of periodontitis and will be helpful in advancing its therapy.

Periodontitis is a process of inflammation in the gingival tissue, which involves the cross talk of multiple cell types. Here, we used CellPhoneDB (55) to profile the communication among cell types in healthy and periodontitis tissues (**Figure 6C**). In both healthy and periodontitis tissues, myeloid cell and nonimmune cells including epithelial, endothelial, and fibroblast cells showed the strongest interactions. While the overall cell-cell interaction increased in the periodontitis tissue, the interaction between myeloid and other immune cells (T, B, and NK cells) exhibited a significant increase. We showed the top 10 cell-cell interactions increased in patients (**Supplementary Figure S4D**). Myeloid and B cells interact more frequently with other cells. Multicolor IHC staining of periodontal tissues showed the increased cell-cell interactions between macrophage (CD11b^+^) and T cells (CD3^+^) and macrophage (CD11b^+^) and B cells (CD19^+^) (**Figures 3D, E** **)**. IL-1, tumor necrosis factor alpha (TNF-α), and interferon gamma (IFN-γ) are important cytokines which are highly involved in the progression of periodontitis (56). We revealed the cell subtype interaction based on the increased expression of these cytokines and their receptors in periodontitis. For instance, M2_cDC1_BATF3 constitutes a major source of IL-β. TNF is mainly derived from Macro_NLRP3 and CD8_GZMK T cells. We noticed that CD8 T cell is the primary source of IFN-γ which interacted with both epithelial and stromal cells. Furthermore, NLRP3^+^ macrophages and CD8 T cells are recruited through CXCR3_CCRL19, which are associated with Fibro_1 and Mural cell. Notably, we also found that CXCL13^+^ fibroblast interacts with B cells through CXCL13-CXCR5 axis, further substantiating its role in immune regulation.

## Discussion

Here, we present the transcriptomic profiling of a total of 29,967 single cells of human gingival tissue using the scRNA-seq method. By identifying an intriguing catalog of cell types and their phenotypes, revealing the altered gene expression profiling and cell-cell communication under diseased condition, our data highlight key areas for advances in the biology of periodontitis that will be helpful in the diagnosis and treatment of periodontitis.

Ten major cell types with great diversity and heterogeneity were identified in our dataset. Junctional epithelial forms the direct attachment to the tooth surface and is exposed to the tooth-adherent microbial communities. We identified the junctional epithelial population with high expression level of SAA proteins. Studies have shown SAA protein triggers inflammatory cytokine secretion *via* interacting with TLR2 pathway in human gingival fibroblasts (57) and the SAA-TLR axis plays an important role in the chronicity of periapical inflammation (58). Thus, our result indicated that SAA-TLR axis in junctional epithelial may be closely related to the first immune-defense mechanisms against periodontal microbiota. Potential regulators of junctional epithelial, such as RUNX2, were identified through SCENIC analysis. This result is in consistency with recent studies where a critical role of RUNX2 in maintaining the integrity of the dentogingival junction was found (59, 60).

Cells, especially immune cells, given the cellular plasticity, have been reported to acquire altered phenotype *in situ* upon specific stimuli within the local biochemical and mechanical microenvironment, which is defined by growth factors, neighboring niche cells, and extracellular matrix (10, 11). Here, in this study, we also found a series of proinflammatory stromal cell subtypes and immune cells that respond to local inflammatory stimuli. We showed the presence of the recently identified antigen-presenting endothelial cells (40) in periodontitis, as revealed by the combined expression of CD31 and MHC class II genes.

Gingival fibroblasts have long been recognized as a heterogeneous population, but the extent of heterogeneity has hitherto remained poorly explored (61). We identified CXCL13^+^ fibroblast subset potentially involved in immune response, which exhibited increased presence in periodontitis. Indeed, CXCL13 is involved in the pathogenesis of several autoimmune diseases and inflammatory conditions by regulating lymphocyte infiltration within the microenvironment (62). Moreover, CXCL13^+^ fibroblast subset was also characterized by high expression of IL-32. Recent evidence has shown that IL-32 activates typical cytokine signal pathways of NF-κB and p38 MAPK. Its expression is closely correlated with proinflammatory cytokines production (TNF-α and IL-1β) and with clinical conditions of periodontitis (63, 64). Furthermore, the enhanced interaction of CXCL13^+^ fibroblast and B cells through CXCL13-CXCR5 were revealed, implying a potential treatment target in periodontitis.

Heterogeneity of myeloid cells was depicted, and novel phenotypes of macrophage, hitherto considered dichotomous (65), were revealed. Our analysis confirmed that the *in vitro*-characterized M1 and M2 cells do not reproduce the given tissue featured with a distinct local environment (66). The increased proportion of CD11b^+^ NLRP3^+^ macrophages in gingival tissue in periodontitis suggested their crucial role in the pathology of periodontitis. Our result also showed an increased level of CCR5 ligand in cytotoxic CD8 T cells of patients, underscoring their role in inflammatory cell recruitment in periodontitis. Chemokine receptor CCR5 is involved in the migration of leukocyte subpopulations throughout experimental periodontitis (67); our result provided further evidence for arresting periodontitis progression with the blockage of CCR1 and CCR5 (68).Taken together, these findings provided new perspectives in the host modulation therapy of periodontitis.

Finally, we further investigated the biology of periodontitis in three directions: profiles of known periodontitis-related genes; comparison between healthy and periodontitis group; and the cell-cell communication alteration between conditions. The increased cell-cell interaction between macrophage and T/B cells in periodontitis highlighted the importance of macrophage in linking the innate and adaptive immune responses and thus in the pathogenesis of periodontal diseases (69, 70). Furthermore, the interaction of NLRP3^+^ macrophages and structural cells through TNF-TNFRSF1A and CXCR3_CCRL19 offered us a hint of the biological function of NLRP3^+^ macrophages in periodontitis.

In gingiva, an important soft tissue within the periodontium, the number of epithelial cells is far more than the number of immune cells. Under pathological conditions, cell types are not affected equally in the development of periodontitis. Thus, scRNA-seq becomes the most impartial and effective approach to obtain the transcriptome of each cell type in the gingiva. Our single-cell profiles not only provide an abundance of resources on the inflammatory responses and cell population dynamics within the microenvironment in periodontitis but also offer insights into the biological foundation of periodontal pathogenesis, which potentially serve as the basis of host modulation therapy.

It is worth noting that we enrolled patients with stage III or IV periodontitis according to the new classification scheme proposed at the 2017 World Workshop on the Classification of Periodontal and Peri-Implant Diseases and Conditions. Due to the fact that no evidence of rapid bone loss could be found, all of the patients were diagnosed with moderate rate of progression (grade B). Our results could serve as a community resource with focus on stage III or IV—grade B periodontitis. Admittedly, the complexity of the periodontitis could not be fully grasped, as the gingiva was located in restricted areas and the number of samples that were sequenced was also limited. Nevertheless, given the robustness of scRNA-seq, it is possible to scale up the current study to provide much improved resolution in the future. In addition, further *in vivo* and *in vitro* mechanistic studies are also needed to verify cell-specific functionality and crucial signaling pathway involved in periodontitis pathogenesis and treatment.

## Data Availability Statement

The sequencing data have been deposited in the China National Genebank Database under the accession number (CNP0001395).

## Ethics Statement

The studies involving human participants were reviewed and approved by the Ethics Committee of Shanghai 9th People’s Hospital in China. The patients/participants provided their written informed consent to participate in this study. Written informed consent was obtained from the individual(s) for the publication of any potentially identifiable images or data included in this article.

## Author Contributions

HL and BL conceived the project. S-jQ, Q-rH, YZ, and L-yZ participated in the data analysis. RC and JM advised the data analysis. S-jQ, L-yZ, and R-yC collected the human donor gingiva and performed the phenotyping and dissection. Q-rH, J-jM, and L-yZ prepared the nuclei sample and performed the single-nuclei RNA-seq. S-jQ, L-yZ, and R-yC performed functional validation. S-jQ, Q-rH, H-cL, and BL wrote the manuscript with input from all other authors. All authors proofread the manuscript. All authors contributed to the article and approved the submitted version.

## Funding

This study has been supported by Shanghai Sailing Program (20YF1423200), the Interdisciplinary Program of Shanghai Jiaotong University (YG2021QN61), the Interdisciplinary Fund (JYJC202110) and the Project of Biobank from Shanghai Ninth People’s Hospital (YBKA201906).

## Conflict of Interest

The authors declare that the research was conducted in the absence of any commercial or financial relationships that could be construed as a potential conflict of interest.

## Publisher’s Note

All claims expressed in this article are solely those of the authors and do not necessarily represent those of their affiliated organizations, or those of the publisher, the editors and the reviewers. Any product that may be evaluated in this article, or claim that may be made by its manufacturer, is not guaranteed or endorsed by the publisher.
