# Supplementary material for: Single-Cell RNA Sequencing Identifies New Inflammation-Promoting Cell Subsets in Asian Patients With Chronic Periodontitis
Source: Front Immunol. 2021 Sep 8;12:711337. doi: 10.3389/fimmu.2021.711337 (PMC8455889; doi:10.3389/fimmu.2021.711337)
Supplement: Supplementary file 1 [file DataSheet_1.docx]

Single-cell RNA sequencing identifies new inflammation-promoting cell subsets in Asian patients

with chronic periodontitis

**Authors:**

Shu-jiao Qian^1*^, Qian-ru Huang^2,3*^, Rui-ying Chen^1^, Jia-ji Mo^1^, Lin-yi Zhou^1^, Yi Zhao^2,3^, Bin Li^2,3#^, Hong-chang Lai^1#^

^*^Authors contributing equally as co-first authors.

^#^ Correspondence authors. Hong-chang Lai, [lhc9@hotmail.com](mailto:lhc9@hotmail.com) and Bin Li, [binli@shsmu.edu.cn](mailto:binli@shsmu.edu.cn)

^1^Department of Implant Dentistry, Shanghai Ninth People’s Hospital, Shanghai Jiao Tong University School of Medicine; College of Stomatology, Shanghai Jiao Tong University; National Center for Stomatology; National Clinical Research Center for Oral Diseases; Shanghai Key Laboratory of Stomatology, Shanghai 200025, China

^2^Shanghai Institute of Immunology, Department of Immunology and Microbiology, Shanghai Jiao Tong University School of Medicine, Shanghai 200025, China

^3^Department of Thoracic Surgery, Shanghai Pulmonary Hospital, Tongji University School of Medicine, Shanghai 200433, China

**Supplementary Materials**

This file includes：

Supplementary figures 1-4

Supplementary tables 1-3

**Supplementary figure1 Quality control and subgroup marker of periodontal single cell sequencing**

**
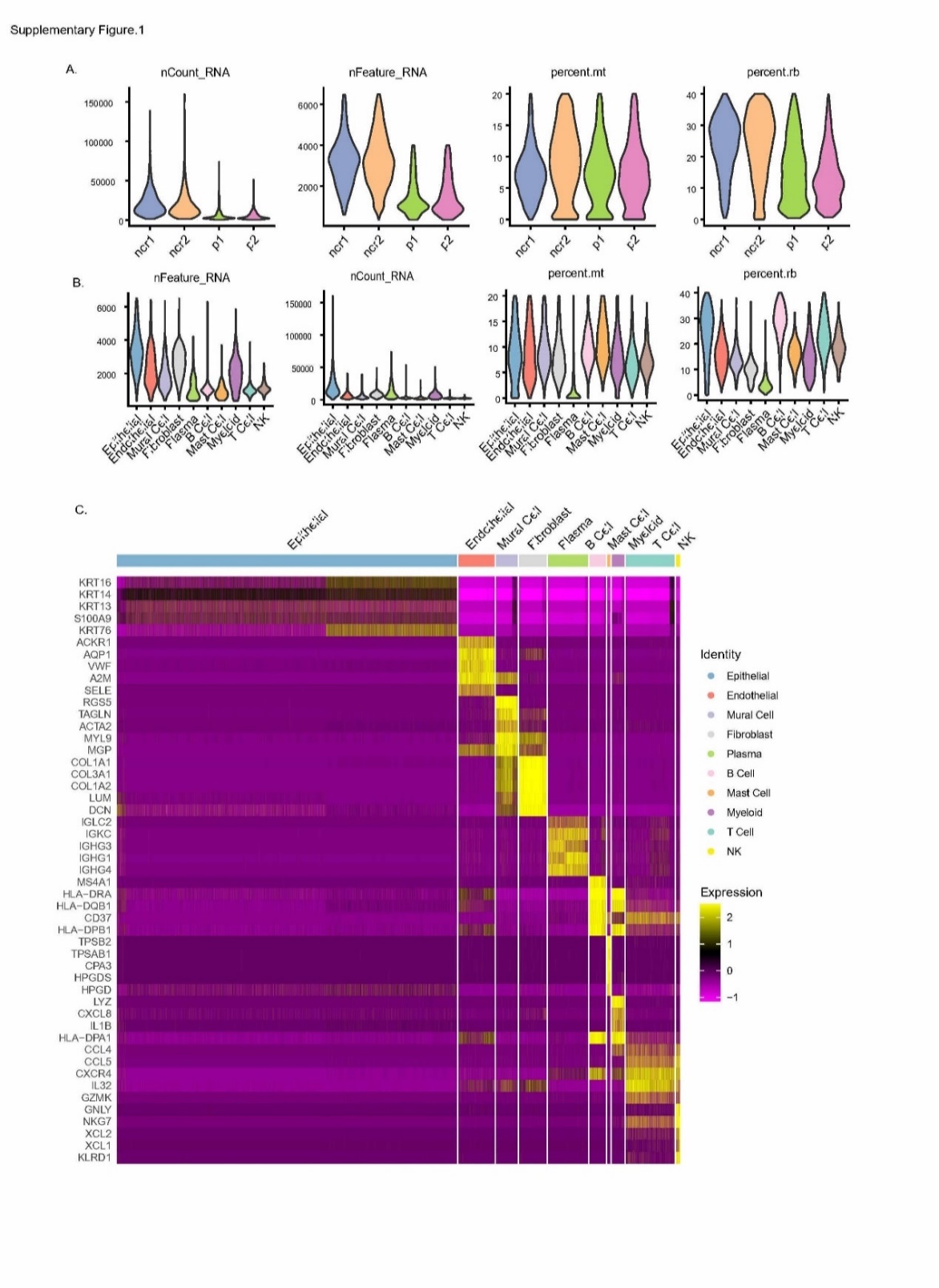
**

1. Violin plots showing the QC metrics (the number of features, RNA counts, percent

mitochondrial transcripts and percent ribosome transcripts) grouped by samples.

1. Violin plots showing the QC metrics (the number of features, RNA counts, percent mitochondrial transcripts and percent ribosome transcripts) grouped by cell types.
2. Heat map showing the top 25 marker gene expression of different cell type.

**Supplementary figure2 The heterogeneity of epithelial compartment**

**
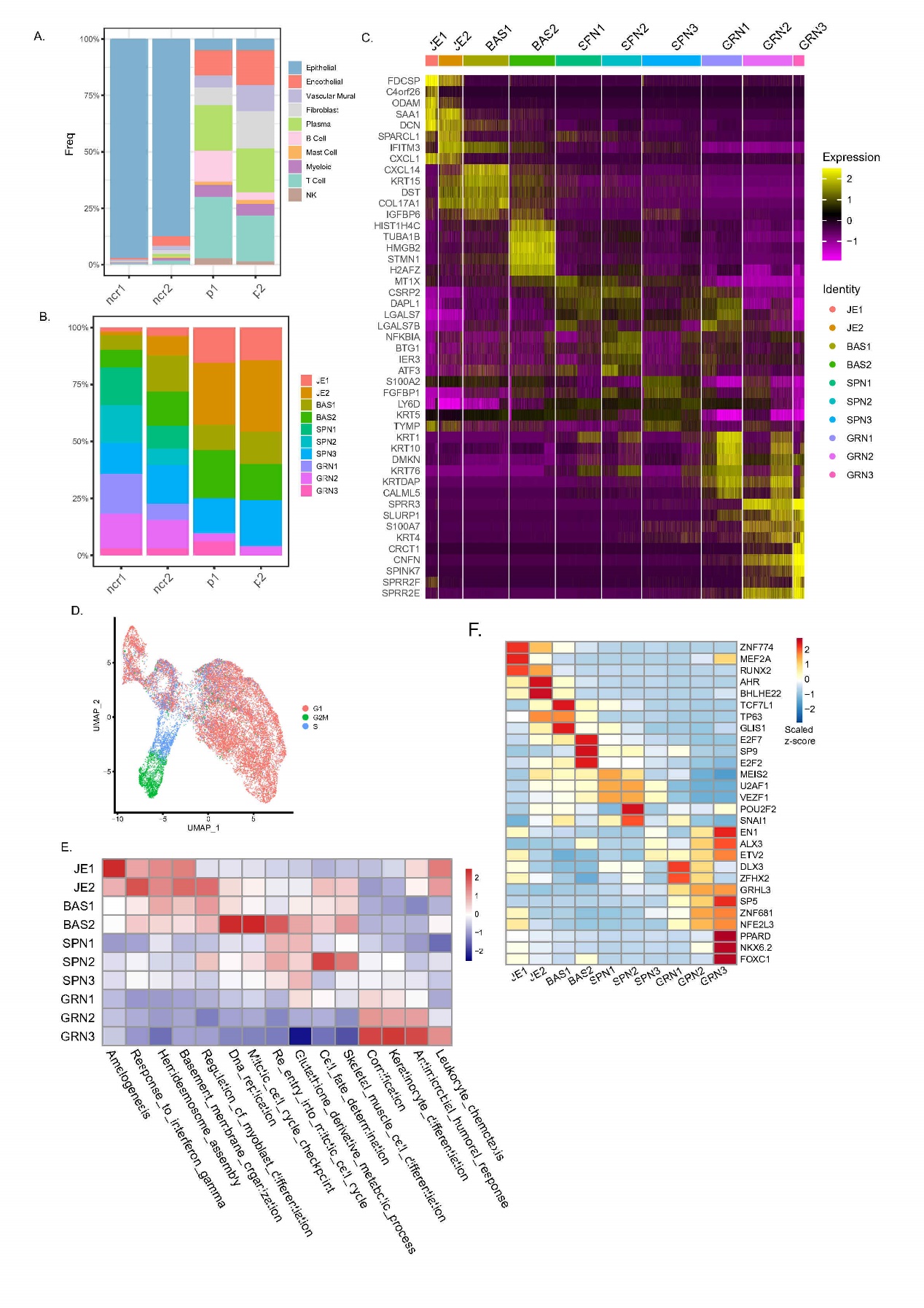
**

1. Proportion of cell types from periodontitis and healthy conditions in Figure 1B (Nor1 and Nor2 are healthy donors; P1 and P2 are periodontitis patients).
2. Proportion of epithelial cell types from periodontitis and healthy conditions in Figure 2A. (Nor1 and Nor2 are healthy donors; P1 and P2 are periodontitis patients).
3. Heat map showing the top5 marker gene expression of ten epithelial cells.
4. Feature Plot shows the score of proliferation signature in epithelial cells.
5. The selected enriched GO terms in different epithelial cell subtypes.
6. Heatmap showing the activity of transcription factors in each epithelial cell subtype. The TF activity is scored using scaled AUCell.

**Supplementary figure3 The heterogeneity of stromal cells**


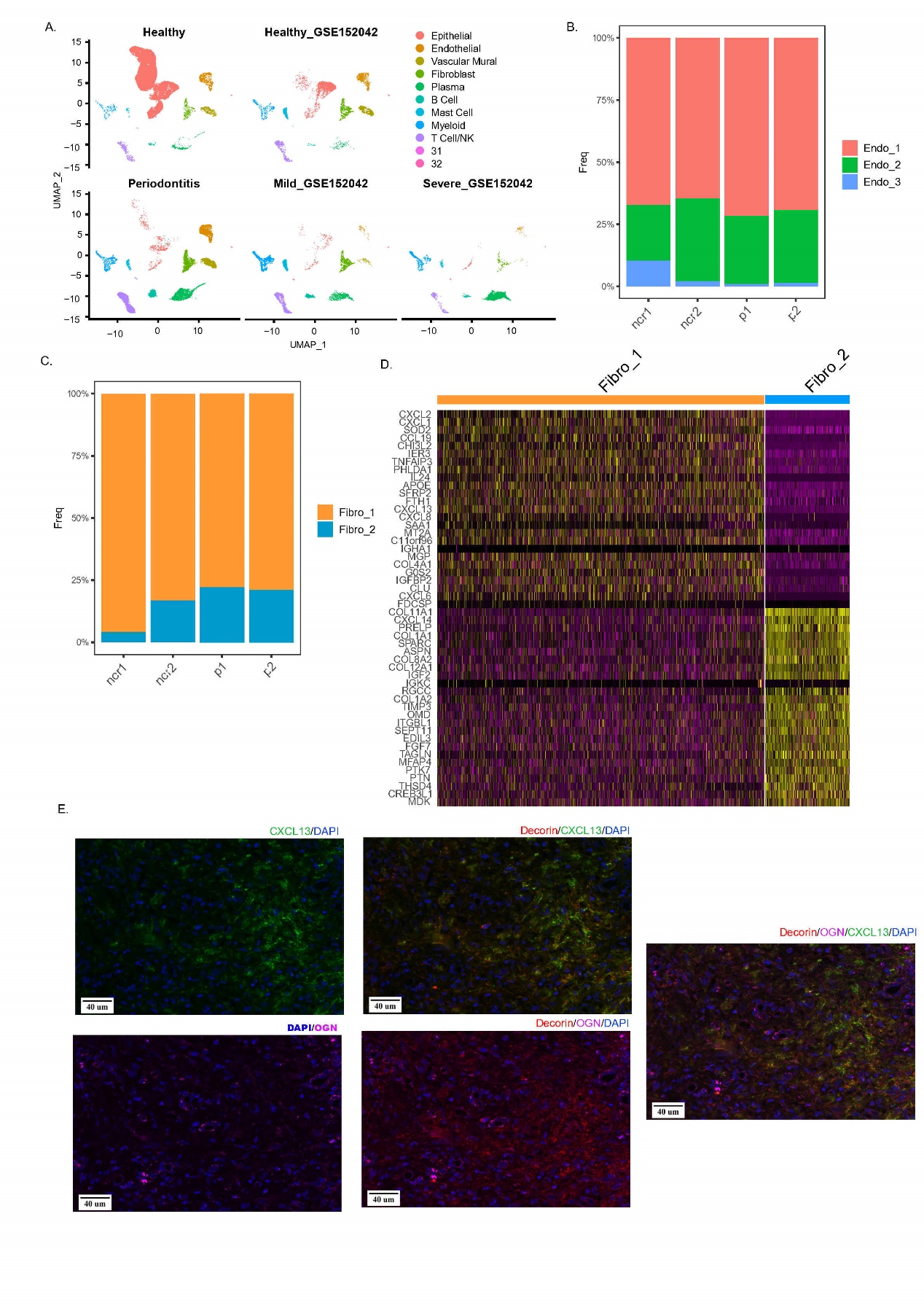


1. Visualization of single cell sequencing data of human gingival cells (combining our data with published data from GSE152042).
2. Proportion of endothelial cells from periodontitis and healthy conditions in Figure 3A (Nor1 and Nor2 are healthy donors; P1 and P2 are periodontitis patients).
3. Proportion of fibroblast cells from periodontitis and healthy conditions in Figure 4A (Nor1 and Nor2 are healthy donors; P1 and P2 are periodontitis patients).
4. Heat map showing the top25 marker gene expression of two fibroblast cells.
5. Immunofluorescent (IF) staining validation of fibroblast subtypes in patient suffering from periodontitis. Red color showed the signal of Decorin staining (fibroblast marker); green color showed the signal of CXCL13 staining; purple color showed the signal of Osteoglycin (OGN) and blue color showed DAPI staining.

**Supplementary figure4 The heterogeneity of immune cells and cell-cell interactions**


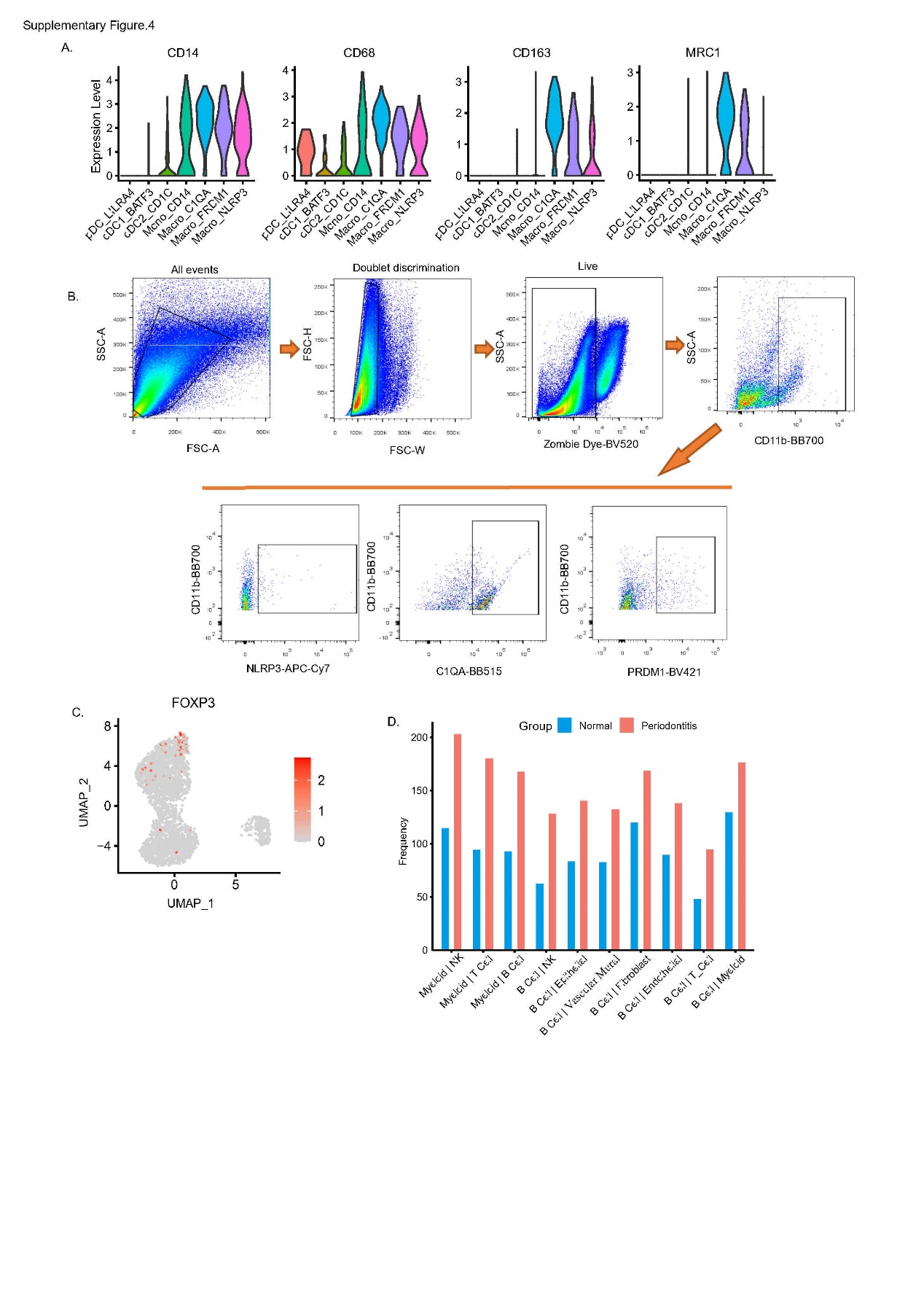


1. Violin plots showing the marker genes for clustering myeloid cells metrics.
2. Example gating strategy for flow cytometry analysis in Figure 5E. We applied a size gate to select for immune cells, another size gate to select single cells, followed by a viability gate (Zombie negative) to exclude dead cells. Live (Zombie-) cells were gated for CD11+ and these were split into NLRP3+, C1QA+ and PRDM1+ cells.
3. Feature Plot shows the expression of FOXP3 in T/NK cells. UMAP displaying the expression of FOXP3 in the T/NK cells.
4. The top 10 cell-cell interactions with the most significant increase in patients compared to healthy participants inferred by CellChat.

**Supplementary Tables**

| **Supplementary Table1A. The demographic information and clinical parameters of patients for scRNA-seq** | | | | | |
| --- | --- | --- | --- | --- | --- |
|  |  | Healthy Group | | Periodontitis Group | |
|  |  | Nor1 | Nor2 | P1 | P2 |
| Sex | | F | F | M | F |
| Age | | 27 | 18 | 46 | 35 |
| Smoking | | no | no | no | no |
| Systemic disease | | no | no | no | no |
| Severity | Interdental clinical attachment loss at site of greatest loss (mm) | 0 | 0 | ≥5 | ≥5 |
|  | Radiographic bone loss | no | no | extending to apical third of the root | extending to middle of the root |
|  | Tooth loss due to periodontitis | no | no | 3 teeth | 1 tooth |
| Complexity | Maximum probing depth (mm) | ≤4 | ≤3 | ≥6 | ≥6 |
| Extent and distribution | | N/A | N/A | generalized | generalized |
| Sample site | Tooth location | 13-23 | 25-27 | 31-42 | 25-26 |
|  | Bleeding on probe | negative | negative | positive | positive |
|  | Plaque score | 0 | 0 | 2-3 | 2-3 |
|  | Probing depth (mm) | 3-4 | 3 | 7-9 | 6-8 |

**Supplementary Table1B. The demographic information and clinical parameters of all patients (mean±standard deviation)**

|  | Healthy Group (N=10) | Periodontitis Group (N=10) |
| --- | --- | --- |
| **Participant characteristic** |  |  |
| Age (mean±SD, yrs) | 38.8±10.01 | 47.2±9.71 |
| Gender (male/female) | 5/5 | 6/4 |
|  | | |
| **Severity and Complexity of periodontitis** |  |  |
| Interdental clinical attachment loss at site of greatest loss (mean±SD, mm) | 0±0.00 | 7.10±1.37 |
| Radiographic bone loss (coronal third/middle third of the root/ apical third of the root) | No | 1/6/3 |
| Tooth loss due to periodontitis (mean±SD) | No | 4.70±1.34 |
| Maximum probing depth (mean±SD, mm) | 3.20±0.42 | 7.50±1.51 |
|  | | |
| **Periodontal measures at sample site** |  |  |
| Probing depth (mean±SD, mm) | 2.88±0.76 | 6.18±1.32 |
| Clinical attachment loss (mean±SD, mm) | 0±0.00 | 5.70±1.51 |
| Plaque index (mean±SD) | 0.12±0.32 | 1.97±0.69 |
| Bleeding index (mean±SD) | 0.32±0.47 | 3.58±0.96 |
| Criteria for periodontal clinical examination: The Bleeding index (Mazza et al., [1981](https://onlinelibrary.wiley.com/doi/10.1111/jcpe.13400#jcpe13400-bib-0033)) ; The Plaque index (Silness & Loe, [1964](https://onlinelibrary.wiley.com/doi/10.1111/jcpe.13400#jcpe13400-bib-0045)) | | |

| **Supplementary Table2. OMIM** | | | | | | | | | |
| --- | --- | --- | --- | --- | --- | --- | --- | --- | --- |
| Cytogenetic location | Gene/Locus | Gene/Locus name | Gene/Locus MIM number | Phenotype | Phenotype MIM number | Inheritance | Pheno map key | Mouse Gene (from MGI) | |
| 6p24.3 | DSP, KPPS2, PPKS2, DCWHKTA | Desmoplakin | 125647 | Dilated cardiomyopathy with woolly hair, keratoderma, and tooth agenesis | 615821 | Autosomal dominant | 3 | Dsp |  |
| 12p13.31 | C1S, EDSPD2 | Complement component-1, s subcomponent | 120580 | Ehlers-Danlos syndrome, periodontal type, 2 | 617174 | Autosomal dominant | 3 | C1s2, C1s1 | |
| 12p13.31 | C1R, EDSPD1 | Complement component-1, r subcomponent | 613785 | Ehlers-Danlos syndrome, periodontal type, 1 | 130080 | Autosomal dominant | 3 | C1rb, C1ra | |
| [4q21.21](https://omim.org/geneMap/4/327?start=-3&limit=10&highlight=327) | ANTXR2, CMG2, HFS | Anthrax toxin receptor 2 | 608041 | Hyaline fibromatosis syndrome | [228600](https://omim.org/entry/228600) | Autosomal recessive | 3 | Antxr2 | |
| [17q24.2](https://omim.org/geneMap/17/828?start=-3&limit=10&highlight=828) | FAM20A, AIGFS, AI1G | Family with sequence similarity 20, member A | [611062](https://omim.org/entry/611062) | Amelogenesis imperfecta, type IG (enamel-renal syndrome) | [204690](https://omim.org/entry/204690) | Autosomal recessive | 3 | [Fam20a](http://www.informatics.jax.org/accession/MGI:2388266) | |
| 2p22.1 | SOS1, GINGF, GF1, HGF, NS4 | SOS Ras/Rac guanine nucleotide exchange factor 1 | 182530 | ?Fibromatosis, gingival, 1 | 135300 | Autosomal dominant | 3 | Sos1 |  |
| 4q12 | REST, NRSF, WT6, GINGF5, HGF5, DFNA27 | RE1-silencing transcription factor | 600571 | Fibromatosis, gingival, 5 | 617626 | Autosomal dominant | 3 | Rest |  |
| 11q14.2 | CTSC, CPPI, PALS, PLS, HMS, PDON1, JPD | Cathepsin C | 602365 | Periodontitis 1, juvenile | 170650 | Autosomal recessive | 3 | Ctsc |  |
| 17q25.1 | ITGB4 | Integrin, beta-4 | 147557 | Epidermolysis bullosa, junctional, non-Herlitz type | 226650 | Autosomal recessive | 3 | Itgb4 |  |

| **Supplementary Table 3** | | | | | |
| --- | --- | --- | --- | --- | --- |
| gene | conMean | otherMean | logFC | pValue | fdr |
| **JE1** |  |  |  |  |  |
| RUNX2... | 0.068459 | 0.006809 | -3.32967 | 0 | 0 |
| ZNF774... | 0.106059 | 0.012 | -3.14379 | 0 | 0 |
| MEF2A... | 0.029666 | 0.00401 | -2.88721 | ####### | ####### |
| MSC... | 0.133465 | 0.01916 | -2.80026 | 0 | 0 |
| HOXC13... | 0.039497 | 0.006598 | -2.58168 | ####### | ####### |
| ZNF311... | 0.028872 | 0.006284 | -2.19997 | ####### | ####### |
| GLIS3... | 0.035343 | 0.007863 | -2.16834 | ####### | ####### |
| TFEC... | 0.095658 | 0.022133 | -2.11167 | 0 | 0 |
| TEAD3... | 0.036854 | 0.00879 | -2.06787 | ####### | ####### |
| MEF2D... | 0.034682 | 0.008277 | -2.06708 | ####### | ####### |
| **JE2** |  |  |  |  |  |
| AHR... | 0.017786 | 0.001526 | -3.54292 | 0 | 0 |
| BHLHE22... | 0.014368 | 0.00143 | -3.32831 | 0 | 0 |
| RUNX2... | 0.053434 | 0.00587 | -3.18633 | 0 | 0 |
| TCF4... | 0.050897 | 0.006452 | -2.97986 | 0 | 0 |
| MYEF2... | 0.06795 | 0.0098 | -2.7936 | 0 | 0 |
| MSC... | 0.113549 | 0.01688 | -2.74995 | 0 | 0 |
| ZNF319... | 0.028154 | 0.004605 | -2.61211 | 0 | 0 |
| ZNF774... | 0.070003 | 0.011458 | -2.61104 | 0 | 0 |
| FOXJ1... | 0.009109 | 0.001526 | -2.57796 | ####### | ####### |
| CREBZF... | 0.065141 | 0.013295 | -2.29273 | ####### | ####### |
| **BAS1** |  |  |  |  |  |
| TCF7L1… | 0.112145 | 0.01735 | -2.69233 | 0 | 0 |
| TP63... | 0.199653 | 0.036259 | -2.46108 | 0 | 0 |
| GLIS1... | 0.048674 | 0.009441 | -2.36618 | 0 | 0 |
| THRB... | 0.034696 | 0.007288 | -2.25124 | 0 | 0 |
| ZNF549... | 0.031147 | 0.006544 | -2.25073 | 0 | 0 |
| KLF15... | 0.027593 | 0.006157 | -2.16391 | 0 | 0 |
| TP73... | 0.150438 | 0.036957 | -2.02525 | 0 | 0 |
| NR2F2... | 0.014203 | 0.003646 | -1.96179 | 0 | 0 |
| NKX2.8... | 0.066802 | 0.017985 | -1.8931 | 0 | 0 |
| TCFL5... | 0.006787 | 0.001903 | -1.83472 | ####### | ####### |
| **BAS2** |  |  |  |  |  |
| E2F7... | 0.031658 | 0.00836 | -1.92096 | 0 | 0 |
| SP9... | 0.096323 | 0.027449 | -1.81113 | 0 | 0 |
| E2F2... | 0.029134 | 0.00844 | -1.78737 | 0 | 0 |
| E2F8... | 0.037544 | 0.011784 | -1.67169 | 0 | 0 |
| NFYB... | 0.026476 | 0.00954 | -1.47266 | 0 | 0 |
| FOXC1... | 0.010179 | 0.003675 | -1.46956 | 0 | 0 |
| POLE3... | 0.045548 | 0.01649 | -1.46578 | 0 | 0 |
| MYBL2... | 0.088204 | 0.032134 | -1.45674 | 0 | 0 |
| E2F1... | 0.021569 | 0.00865 | -1.31823 | 0 | 0 |
| BRCA1... | 0.0412 | 0.017917 | -1.20128 | 0 | 0 |
| **SPN1** |  |  |  |  |  |
| VEZF1... | 0.018965 | 0.008848 | -1.10001 | ####### | ####### |
| MEIS2... | 0.061547 | 0.028741 | -1.09859 | ####### | ####### |
| U2AF1... | 0.026304 | 0.013084 | -1.00754 | ####### | ####### |
| TCF12... | 0.009058 | 0.005536 | -0.71034 | 1.70E-78 | 4.96E-78 |
| HLF... | 0.032727 | 0.020723 | -0.65925 | ####### | ####### |
| RBPJ... | 0.0068 | 0.004342 | -0.64733 | ####### | ####### |
| SETDB1... | 0.003985 | 0.002606 | -0.61265 | 5.33E-68 | 1.39E-67 |
| BCL6... | 0.006509 | 0.004549 | -0.51693 | 3.53E-53 | 7.26E-53 |
| GABPA... | 0.004792 | 0.003428 | -0.48359 | ####### | ####### |
| KLF8… | 0.005985 | 0.004311 | -0.47325 | 9.67E-83 | 2.90E-82 |
| **SPN2** |  |  |  |  |  |
| POU2F2... | 0.02054 | 0.007983 | -1.36349 | 0 | 0 |
| SNAI1... | 0.056554 | 0.022144 | -1.3527 | ####### | ####### |
| VEZF1... | 0.018566 | 0.009082 | -1.03154 | ####### | ####### |
| U2AF1... | 0.026957 | 0.013252 | -1.02446 | ####### | ####### |
| EGR2... | 0.042966 | 0.021752 | -0.98201 | 0 | 0 |
| REL... | 0.041153 | 0.020857 | -0.98048 | 0 | 0 |
| SETBP1... | 0.026011 | 0.013657 | -0.92943 | 0 | 0 |
| TRPS1... | 0.072921 | 0.038492 | -0.92178 | ####### | ####### |
| MSX1... | 0.028599 | 0.015724 | -0.86296 | 0 | 0 |
| FOXN3... | 0.070913 | 0.039758 | -0.83479 | 0 | 0 |
| **SPN3** |  |  |  |  |  |
| EN1... | 0.035839 | 0.027501 | -0.38206 | 3.86E-48 | 5.52E-48 |
| ALX3... | 0.025131 | 0.01958 | -0.36007 | 1.94E-32 | 2.43E-32 |
| ETV2... | 0.040647 | 0.033679 | -0.27131 | ####### | ####### |
| PSMD12... | 0.053925 | 0.044746 | -0.26921 | 0 | 0 |
| POLE4... | 0.060593 | 0.050508 | -0.26264 | ####### | ####### |
| POLR3G... | 0.062185 | 0.052015 | -0.25763 | ####### | ####### |
| RARG... | 0.226386 | 0.189931 | -0.25331 | ####### | ####### |
| MAF... | 0.173767 | 0.150549 | -0.20691 | 7.90E-53 | 1.17E-52 |
| ESRRA... | 0.062795 | 0.05454 | -0.20334 | 0 | 0 |
| NFE2L2... | 0.321274 | 0.282369 | -0.18622 | 9.60E-79 | 1.81E-78 |
| **GRN1** |  |  |  |  |  |
| DLX3... | 0.16602 | 0.043543 | -1.93084 | 0 | 0 |
| ZFHX2... | 0.022807 | 0.007591 | -1.58702 | 0 | 0 |
| GRHL3... | 0.241802 | 0.102567 | -1.23726 | 0 | 0 |
| SP5... | 0.093206 | 0.040101 | -1.21678 | ####### | ####### |
| RORC... | 0.04068 | 0.018323 | -1.15071 | 0 | 0 |
| OLIG3... | 0.060899 | 0.028633 | -1.08875 | ####### | ####### |
| ZNF16... | 0.014041 | 0.006633 | -1.08181 | ####### | ####### |
| DLX5... | 0.100841 | 0.050718 | -0.99151 | 0 | 0 |
| GATA3... | 0.064057 | 0.0324 | -0.98336 | 0 | 0 |
| NFE2L3... | 0.009454 | 0.004812 | -0.97433 | ####### | ####### |
| **GRN2** |  |  |  |  |  |
| SP5... | 0.168707 | 0.026986 | -2.64426 | 0 | 0 |
| ZNF681... | 0.040639 | 0.009417 | -2.10952 | 0 | 0 |
| NFE2L3... | 0.015464 | 0.003756 | -2.04163 | ####### | ####### |
| GRHL3... | 0.325054 | 0.085823 | -1.92125 | 0 | 0 |
| BACH1... | 0.02968 | 0.010125 | -1.55164 | 0 | 0 |
| NKX6.2... | 0.02449 | 0.008743 | -1.48594 | 0 | 0 |
| PPARG... | 0.068109 | 0.02477 | -1.45923 | 0 | 0 |
| PAX6... | 0.057909 | 0.021784 | -1.41056 | 0 | 0 |
| ZNF16... | 0.015149 | 0.006253 | -1.27654 | ####### | ####### |
| RORC... | 0.041334 | 0.01759 | -1.23261 | 0 | 0 |
| **GRN3** |  |  |  |  |  |
| PPARD... | 0.023757 | 0.000901 | -4.72097 | 0 | 0 |
| NKX6.2... | 0.110624 | 0.007782 | -3.82942 | 0 | 0 |
| FOXC1... | 0.037215 | 0.003469 | -3.42347 | ####### | ####### |
| TEAD1... | 0.033747 | 0.003411 | -3.30639 | ####### | ####### |
| ZNF117... | 0.350125 | 0.044663 | -2.97072 | 0 | 0 |
| KDM4B... | 0.015821 | 0.002066 | -2.93669 | ####### | ####### |
| SP5... | 0.284211 | 0.038567 | -2.88152 | 0 | 0 |
| BACH1... | 0.078595 | 0.010711 | -2.87531 | 0 | 0 |
| GLIS3... | 0.045578 | 0.007663 | -2.5723 | ####### | ####### |
| FOXD1... | 0.118499 | 0.020392 | -2.53881 | 0 | 0 |
